# Data quality of whole genome bisulfite sequencing on Illumina platforms

**DOI:** 10.1371/journal.pone.0195972

**Published:** 2018-04-18

**Authors:** Amanda Raine, Ulrika Liljedahl, Jessica Nordlund

**Affiliations:** Department of Medical Sciences, Molecular Medicine and Science for Life Laboratory, Uppsala University, Uppsala, Sweden; Montana State University Bozeman, UNITED STATES

## Abstract

The powerful HiSeq X sequencers with their patterned flowcell technology and fast turnaround times are instrumental for many large-scale genomic and epigenomic studies. However, assessment of DNA methylation by sodium bisulfite treatment results in sequencing libraries of low diversity, which may impact data quality and yield. In this report we assess the quality of WGBS data generated on the HiSeq X system in comparison with data generated on the HiSeq 2500 system and the newly released NovaSeq system. We report a systematic issue with low basecall quality scores assigned to guanines in the second read of WGBS when using certain Real Time Analysis (RTA) software versions on the HiSeq X sequencer, reminiscent of an issue that was previously reported with certain HiSeq 2500 software versions. However, with the HD.3.4.0 /RTA 2.7.7 software upgrade for the HiSeq X system, we observed an overall improved quality and yield of the WGBS data generated, which in turn empowers cost-effective and high quality DNA methylation studies.

## Introduction

Methylation of cytosine residues (5-mC) in the CpG context is a key epigenetic mark which is involved in processes such as regulation of gene expression, cell differentiation, genomic imprinting, X-chromosome inactivation, transposon silencing and chromosomal stability [1]. Aberrant methylation patterns have been shown to be associated with a growing number of conditions and disease, in particular cancer [2].

Bisulfite conversion, in which unmethylated cytosines are converted to uracil (and subsequently to thymine after PCR) whilst methylated cytosines remain unchanged [3], remains the gold standard technique for detecting DNA methylation and is often used in combination with SNP genotyping or next generation sequencing (NGS) for interrogation of DNA methylation of individual CpG sites. The most popular method has been the Infinium BeadChip assays, which interrogate the methylation status of cytosine residues by genotyping cytosine or thymine (methylated vs unmethylated cytosine residues) using a predetermined set of probes in a microarray format. The Infinium assays offer quantitative measurement of DNA methylation and have been launched with increasing numbers of target CpG sites over the last decade [4]. The EpiTYPER-MassARRAY (Agena) is a different type of assay that allows high-throughput quantitative methylation analysis of multiple CpG sites in PCR amplicons in a workflow comprising bisulfite conversion, targeted PCR amplification and MALDI-TOF mass spectrometry [5]. More recently, direct read-out of methylation status of single CpG sites and other base modifications has been demonstrated using new single-molecule sequencing chemistries that circumvent the need for chemical modification of DNA for example on the PacBio or Oxford Nanopore platforms [6, 7].

However, whole genome bisulfite sequencing (WGBS) using massively parallel short-read sequencing is the only method widely available today that can provide an affordable, high throughput and unbiased view of the entire methylome, which comprises ~28 million CpG sites in humans. Bisulfite converted sequencing-libraries constitutes a particular case of low diversity sequences where the base composition is reduced to virtually three nucleotides (A,T,G) and a very small fraction of Cs, which represents the small portion of methylated cytosines in a genome. Sequencing of libraries with unbalanced base composition on the Illumina systems has historically been challenging, frequently leading to low data yields and inferior sequencing quality [8] [9] [10]. Such issues can, in part, be attributed to earlier versions of the software operating the image analysis and base calling onboard the instrument, which were not adapted for handling low diversity sequences. The HiSeq Control Software (HCS) operates the imaging and calls the Real Time Analysis software (RTA) to execute intensity extraction, base calling and quality scoring. Previously, a dedicated control lane with a balanced library with A/T and G/C equally represented and a high level of spike-in with a high diversity library such as PhiX was required to improve data quality and yield [10]. For WGBS specifically, low base calling quality scores (Q-scores) assigned to guanines (representing methylated positions) have been reported in data generated with a certain version of HCS and RTA on the HiSeq 2500 system [11]. Moreover, the aforementioned WGBS data was shown to significantly deviate in terms of global methylation levels compared to data generated from identical libraries using other HiSeq 2500 software versions on the same instrument, suggesting that the reduced Q-scores of nucleotides representing 5-mC in bisulfite sequencing may result in biased methylation levels. Updates of the HCS and RTA software, which were first implemented on the MiSeq system and later also on the HiSeq2500 system (from HCS v2.2.38/ RTA 1.18.61 and forward), conferred significant improvements in data yield and sequence quality of low diversity libraries, including bisulfite converted libraries [10].

Illumina’s HiSeq X system is the current work horse for whole genome sequencing studies. The patterned flow cell technology on the HiSeq X has significantly increased throughput and pushed costs close to the $1000 genome. The HiSeq X platform is now open to WGBS at an equal favorable price per base, and thus holds great promise for large-scale methylome studies. However, considering previous software related issues with bisulfite sequencing, an examination of the quality of WGBS data generated on the HiSeq X system is warranted.

We therefore performed a retrospective quality control analysis of WGBS data that was generated from a set of control DNA samples within our core facility over time using different software versions on the HiSeq X, the HiSeq2500 and most recently, the NovaSeq. We identified substantial low Q-scores assigned to guanines in the second read of paired-end WGBS data with certain HCS/RTA versions. Notably, this issue was mitigated in the most recent HiSeq X software update HCS: HD.3.4.0 /RTA 2.7.7 and we demonstrate that the latest HCS/RTA version provide sequences of high quality, comparable to WGBS data generated on the HiSeq 2500 system. Despite low Q-scores assigned to guanines by certain software versions, we observed only minor differences in global methylation levels across libraries prepared with the same method. Rather, we observe that global methylation rates vary more depending on the choice of library preparation protocol. In spite of differences in global methylation rates, correlation of methylation at individual CpG sites across methods and sequencing software were in general high.

## Results and discussion

Low Q-scores assigned to guanines in bisulfite sequencing reads generated with HiSeq 2500 systems installed with certain HCS/RTA software versions have previously been observed [11]. This particular issue was primarily observed at guanine positions in the second read (R2) of directional MethylC-Seq libraries whereas the first read (R1) did not significantly suffer from low Q-scores at any nucleotide type. The small fraction of cytosines, which are not converted during bisulfite treatment, represents methylated positions in the genome. Most types of WGBS library protocols are designed to sequence the original top and bottom DNA strands and thus methylated positions are sequenced as cytosines in R1 and as guanines in R2 of paired-end sequencing. Interestingly, a recent study demonstrated low Q-scores at guanines in R1 for PBAT-type bisulfite sequencing libraries, in a HCS/RTA software version specific manner [12]. In contrast to the majority of WGBS library protocols, PBAT libraries are constructed to sequence the complementary to the original strands, hence methylated cytosines are sequenced as guanines in R1 and as cytosines in R2 [12]. Low Q-scores assigned to guanines in R2 of MethylC-seq libraries or in R1 of PBAT libraries implies that the issue is related to low content of guanines in bisulfite converted reads. Moreover, as the fraction of guanines in R2 of most WGBS libraries (or R1 of PBAT) is very low, extensive Q-score reduction at guanines will not be obvious when inspecting average base quality in general. Overall low Q-scores of guanines in WGBS data may indicate a higher probability of base calling errors, which in turn could cause technical variance of methylation levels. Moreover, reduced Q-scores could potentially lead to lower alignment rates and data loss as less data might pass quality filtering. For bisulfite sequencing on HiSeq 2500 (v4 chemistry), the guanine Q-score issue was mitigated from software update HCS v2.2.38/RTA 1.18.61 and forward so that WGBS data generated with this system is now generally of high quality [13] and can such be used for comparison with data generated on the HiSeq X and NovaSeq systems.

In order to retrospectively examine the quality of bisulfite data generated with the HiSeq X system we analyzed a set of WGBS libraries from a core set of DNA samples that were prepared with three different library preparation protocols and sequenced across different software versions. The WGBS data was generated with three different HCS and RTA versions for HiSeq X; HCS v3.3.39/RTA 2.7.1, v3.3.75/RTA 2.7.5, and the most recent update; HD.3.4.0 /RTA 2.7.7. In addition we included WGBS data generated from a pilot sequencing run on the NovaSeq instrument. For this comparison we examined sequence data that were generated from Accel-NGS Methyl Seq (Swift Biosciences), TruSeq DNA Methylation (TSDM, formerly EpiGnome, Illumina Inc) and splinted ligation adapter tagging (SPLAT, [14]) libraries prepared from lymphoblastoid cell lines (NA10860, NA11992) or the leukemic cell line REH The DNA samples used, library pools and sequence data generated is outlined in S1 Fig. General metrics for all of the sequencing runs are found in Table 1. For comparison we included previously published data generated from libraries from the same DNA samples sequenced on the HiSeq 2500 system (HCS v2.2.38/RTA 1.18.61) [13]. Hereafter all versions of HiSeq or NovaSeq software will be presented by the RTA version alone.

**Table 1:** Per library sequencing metrics.

Library	Sample	# read pairs^a^(M)	% Alignment rate^a^	% Duplicate reads	Gb seq	Gb yield^b^	% yield^b^	Insert size	% PhiX
*HiSeqX HCS*: *HD*.*3*.*4*.*0 RTA*: *2*.*7*.*7*									
Accel-1a	NA10860	501	78–79	15–20	150	78	52	175	2
SPLAT-1a	NA10860	470	79–80	16–21	142	84	59	185	2
TSDM-5	NA10860	550	77–79	38–40	166	66	40	167	2
TSDM-6	REH	506	77–79	34–37	153	64	42	170	2
TSDM-7	REH	303	78–80	36–38	93	37	40	154	2
*HiSeqX HCS*: *3*.*3*.*39 RTA*: *2*.*7*.*1*									
SPLAT-1a	NA10860	83	75	11	24	15	62	185	≥ 20
SPLAT-1b	NA10860	191	75	13–14	58	33	57	178	≥ 20
SPLAT-3a	REH	81	73	11	25	14	58	188	≥ 20
SPLAT-3b	REH	174	74–75	12–14	53	13	59	195	≥ 20
TSDM-1a	NA10860	80	68	13	24	11	46	158	≥ 20
TSDM-2	REH	52	73	17	26	10	41	143	≥ 20
*HiSeqX HCS*: *3*.*3*.*75 RTA*: *2*.*7*.*5*									
SPLAT-5	NA11992	184	69–71	14–17	38	20	53	204	≥ 20
SPLAT-6	NA11992	119	70–73	14–18	36	20	55	209	≥ 20
SPLAT-7	NA11992	115	69–72	14–18	35	19	54	210	≥ 20
SPLAT-8	NA10860	128	73–76	14–17	39	21	54	160	≥ 20
SPLAT-9	NA10860	128	68–71	15–19	39	21	54	191	≥ 20
TSDM-3	NA10860	145	78	20	44	21	48	140	≥ 20
TSDM-4	REH	117	79	14	35	19	52	144	≥ 20
*NovaSeq Control Software 1*.*0*.*2 RTA*:*3*.*1*.*5*									
TSDM-7	REH	287, 282	70, 70	46, 46	170	48	28	143	> 50
*HiSeq2500 HCS*: *2*.*2*.*38 RTA*: *1*.*18*.*61*									
SPLAT-10ab	NA10860	342	70	1	79	51	64	198	10
SPLAT-11ab	REH	362	80	1	90	68	75	193	10
Accel-1ab	NA10860	318	82	1	79	52	66	173	10
Accel-2ab	REH	266	82	1	66	43	65	168	10
TSDM-1a	NA10860	342	77	15	85	46	54	159	10
TSDM-8	REH	355	78	13	88	51	58	157	10

^a^ reported as the total number of reads across lanes per library

^b^ Yield is defined as the number of Giga bases retained after pre-processing, mapping and deduplication^.^

### Low quality scores assigned to guanines in R2 by certain HiSeq X HCS/RTA software versions

**Fig 1:**
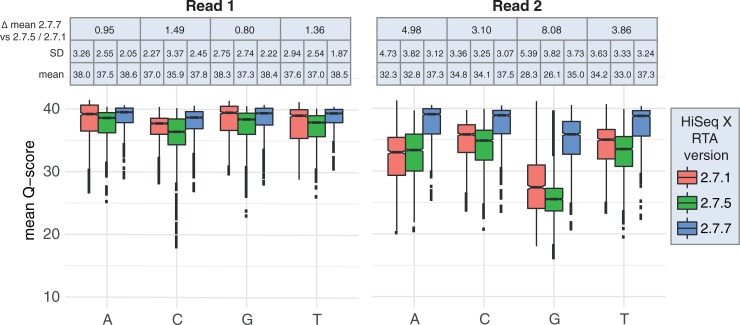
Box plots of the mean per-base Q-scores for R1 (left) and R2 (right) across three HiSeq X RTA versions. The mean, standard deviation (SD), and mean overall difference in Q-scores between RTA 2.7.7 and the two older versions (RTA 2.7.1 and 2.7.5) are listed above the box plots for each base and RTA version.

**Fig 2:**
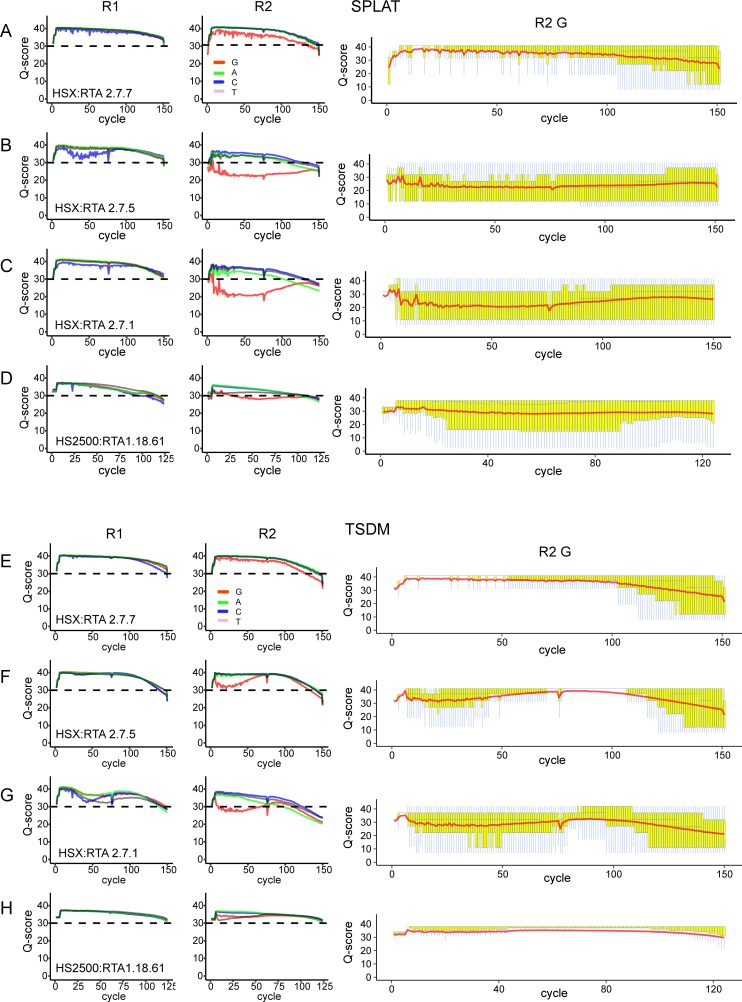
Examples of average base call quality scores for whole genome bisulfite sequencing of libraries prepared from lymphoblastoid cell line NA10860. Per nucleotide quality scores (average for each sequencing cycle) for read 1 and read 2 separately. A-D) SPLAT libraries. E-H) TSDM libraries. Panels A,B,C and E,F,G show Q-scores obtained with HiSeq X RTA versions, the version numbers are noted in each panel. Panels D and H show corresponding data generated on the HiSeq 2500 platform. Q-boxplots for guanines exclusively in read 2 are plotted in the rightmost panels.

**Fig 3:**
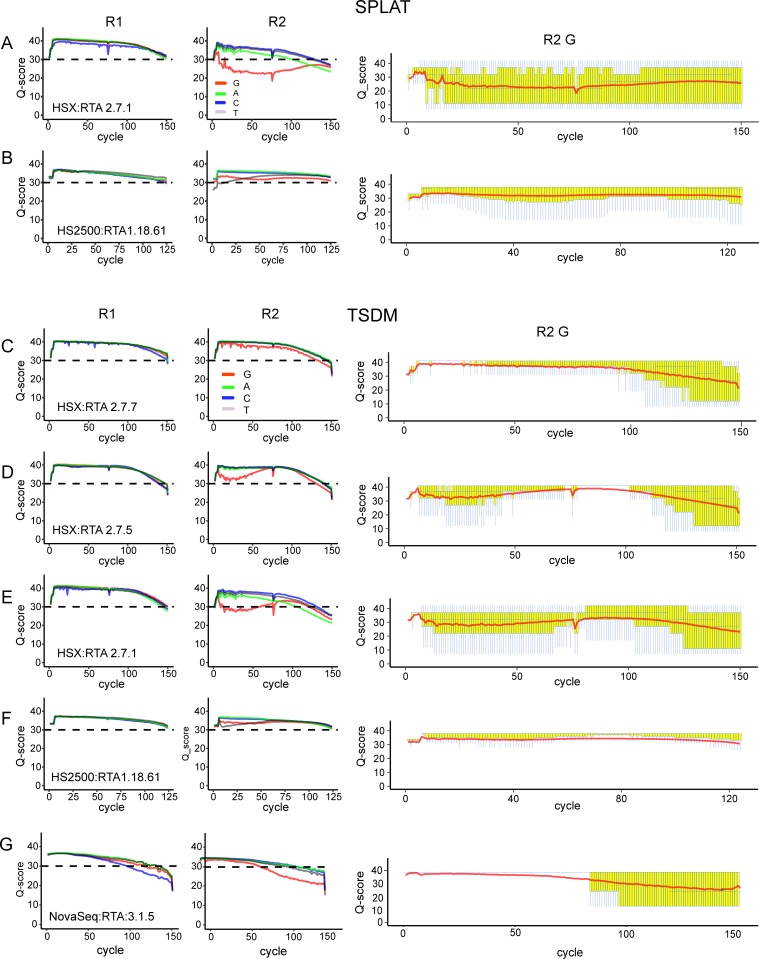
Examples of average base call quality scores for whole genome bisulfite sequencing of libraries prepared from leukemia cell line REH. Per nucleotide quality scores (average for each sequencing cycle) for read 1 and read 2 separately. A and B): SPLAT libraries. C-F) TSDM libraries. HiSeq X RTA versions are plotted in Panels A, and C-E. Corresponding data generated on the HiSeq 2500 are plotted in panels B and F and data generated on the NovaSeq are plotted in panel G. Q-boxplots for guanines exclusively in read 2 is plotted in the rightmost panels.

To assess the quality of data generated by different HCS/RTA versions on HiSeq X we considered Q-scores assigned to the four nucleotide types separately (A, C, G and T) on three HiSeqX versions: RTA 2.7.1, RTA 2.7.5, and RTA 2.7.7. For each of the four bases, in R1 and R2, RTA 2.7.7 displayed significantly higher Q-scores than the previous two version (Fisher’s exact p-value < 0.001, Fig 1), showing an overall improvement in the quality scoring. For all bases in R1 high Q-scores (>30) were still assigned to each of the four nucleotide types (average Q-score per nucleotide type: 33–38), irrespective of sample, library protocol, or software version on the sequencer (S1 and S2 Tables). The overall differences in Q-scores between RTA versions 2.7.1 and RTA 2.7.5 and the newest RTA version 2.7.7 generally were negligible (mean difference across library types: 0.80–1.49). The Q-scores for R2 for all bases and software versions were generally lower and more variable than in R1 (Fig 1). The R2 Q-scores for versions 2.7.1/2.7.5 were significantly lower (Fisher’s exact p-value <0.001) and more variable than in version 2.7.7. Despite this, the Q-scores for adenine, cytosine, and thymine bases were still on average > 30. However, the Q-scores for guanines in R2 were substantially lower in versions 2.7.1/2.7.5 compared to version 2.7.7 (overall difference in Q-score 8.08), even though high amounts of a balanced PhiX library was spiked-in to each lane (20–40%). This feature was observed in all library types and samples, however it was particularly pronounced in data from SPLAT libraries (average G-base Q-scores: 22–29) in comparison to data generated with TSDM libraries (average: 26–32) (Figs 2 and 3, S1 Table). SPLAT libraries prepared from various human sample sources (REH, NA10860, NA11992), each with differing levels of global methylation, were equally affected (S1 Table). TSDM libraries display a typical and pronounced GC bias (average 26.1–26.8% GC content in bisulfite converted reads for NA10860), meaning that GC regions are overrepresented in this library type, whereas SPLAT libraries have a more even representation of the genome average (22.5–25.1% GC content in bisulfite converted reads for NA10860) [13]. Therefore, it appears that the guanine Q-scores assigned by RTA: 2.7.1 and RTA: 2.7.5 appear to improve with increasing GC content of the library (S2 Fig).

### WGBS data quality and yield with the HiSeqX HD.3.4.0/RTA 2.7.7 software update

The HCS/RTA software update for the HiSeq X system (HD.3.4.0 /RTA 2.7.7) was released by Illumina in February 2017 in order to achieve better performance of WGBS on the HiSeq X. For this software version, WGBS data was available for three different WGBS library types (TSDM, SPLAT and Accel-NGS) to an average of ~ 500 M read pairs per library with a 2% PhiX spiked-in (S1 Fig).

Generally, all the libraries displayed high Q-scores for all four nucleotides types in both reads pairs, which were consistent across all sequencing lanes at similar levels to WGBS data generated from the same library types and DNA samples on the HiSeq 2500 (Figs 2 and 3, S3 Fig and S2 Table). Notably, the same exact SPLAT library that resulted in low Q-scores when sequenced with RTA 2.7.1 (SPLAT-1a, average guanine Q-score:22) exhibited much improved quality when the same library was sequenced with software version RTA 2.7.7 (SPLAT-1a, average guanine Q-score: 32–33). This finding strengthens the notion that the Q-score issue is software related and does not originate from the sequencing library per se. Moreover, the trend towards higher guanine Q-scores with libraries with increased GC content, which was observed with the earlier HiSeq X software versions, was not as evident in the data generated by software version RTA 2.7.7 (S2 Fig).

Next, we were interested in how the amount of “usable” data (post alignment) obtained from a HiSeq X lane compares to a HiSeq2500 lane. Alignment rates of the WGBS data generated on the HiSeq X with RTA 2.7.7 were on par with previously generated WGBS data from HiSeq 2500 [13] and higher than those obtained from the earlier HiSeq X software version (77–80% as compared to 65–75%) (Table 1). The levels of duplicate reads were 15–20% for SPLAT and Accel-NGS Methyl-Seq, whilst the TSDM libraries had higher (34–40%) duplication rates. All of the libraries displayed higher duplication rates on the HiSeq X than what was observed for the same library types sequenced on HiSeq 2500 (~2% and 15% for SPLAT and TSDM respectively). A large part of the duplicated reads in the SPLAT and Accel-NGS libraries are likely ExAmp duplicated reads, although we did not observe any obvious difference in the level of duplicate reads when loading different amounts of library of on the flow cell (100, 150 or 200 pM) (Table 1). Following adapter and quality trimming, mapping and deduplication, 57–61% of the generated raw data was retained for the SPLAT library, 50–52% for the Accel-NGS Methyl-Seq library and 34–40% for the TSDM libraries (Table 1). Thus, more data was retained for SPLAT and Accel-NGS Methyl-Seq libraries, as a result of longer insert sizes and lower duplication rates compared to the TSDM libraries. On average we obtained data (post mapping and deduplication) corresponding to ~25x genome coverage per HiSeq X lane for SPLAT, ~22x for Accel-NGS Methyl-Seq, and ~17x for TSDM. Thus from one HiSeq X lane we generated approximately the same amount of high quality WGBS data as was previously obtained from two HiSeq2500 lanes [13], at approximately one fourth of the cost.

### WGBS on NovaSeq; results from a pilot experiment

NovaSeq is the latest iteration of Illumina sequencers for which the patterned flow cell technology is paired with a 2-color detection system for enhanced speed and throughput. For an initial assessment of WGBS data generated on the NovaSeq system we ran a single TSDM WGBS prepared from the REH cell line (TSDM-7), which was also sequenced on the HiSeqX with RTA: 2.7.7. The library was sequenced on a single S2 flow cell across two lanes together with a pool of well-balanced libraries corresponding to 12% of the data derived from each lane. The remaining fraction contained PhiX (50%, installation run) and RNA-seq libraries. We observed a tendency towards lower Q-scores at the end of reads, which was most prominent for the methylated bases (C in R1 and G in R2, Fig 2G). The alignment rate was lower (70%) than obtained for the identical TSDM-7 library sequenced on HiSeqX (80%), most likely as a consequence of the lower Q-scores (Table 1). High duplication rates and lower mapping efficiencies resulted in a total yield of 28% of the unprocessed data compared to 40% for the identical TSDM-7 library sequenced on HiSeqX (RTA2.7.7).

### Minor variation in average methylation levels between data derived from different HCS/RTA software versions

**Fig 4:**
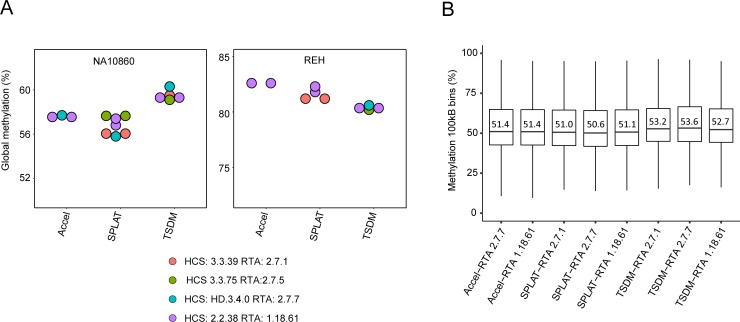
Variation in global methylation rates depends less on RTA version than on library preparation method. A) Global methylation levels (average methylation level across the whole genome) for DNA samples NA10860 and REH are shown for the different library preparation methods and are colored according to RTA software. B) Boxplots showing the average methylation in 100 kB windows for the various libraries and RTA versions; the median values are denoted in the panel.

In a previous study, up to 5% deviation in global methylation levels was observed in PBAT WGBS libraries sequenced with HCS 2.0.12/RTA 1.17.21.3 version on the HiSeq 2500, presumably due to low Q-scores of guanine bases that correspond to methylated cytosines [11]. Thus, in a similar fashion, we compared global methylation levels across data generated on the HiSeq X with the various software versions and HiSeq 2500 in order to determine if the low Q-scores is associated with bias in methylation-calling (Fig 4A, S3 Table). Notably, within the same DNA sample and library type we observed only negligible (min-2%) differences in global methylation levels, which does not appear to be software-specific. For example, the largest difference observed was 1.6% between SPLAT-NA10860 libraries produced with the original protocol, sequenced on the HiSeqX RTA 2.7.7 in comparison to the HiSeq2500 (S3 Table). For SPLAT libraries amplified with a different PCR polymerase (Phusion U) the largest difference observed was 2%. As discussed previously, the lowest guanine Q-scores in R2 were observed in SPLAT libraries when sequenced on RTA 2.7.1. Thus, we analyzed the global methylation levels of R1 and R2 independently in these libraries, and found that the largest difference observed was only a 0.5% difference in global methylation (Table 2).

**Table 2:** Global methylation levels computed from R1 and R2 separately.

Library	Sample	Software version	Average methylation R1	Average methylation R2	SR Alignment rate R1	SR Alignment rate R2	PE Alignment rate
SPLAT-1a	NA10860	HCS: HD.3.4.0 RTA:2.7.7	55.5%	55.8%	86.1%	83.7%	80.3%
SPLAT-1a	NA10860	HCS:3.3.39 RTA:2.7.1	55.6%	56.1%	82.5%	77.4%	75.3
SPLAT-1b	NA10860	HCS: 3.3.39 RTA:2.7.1	55.6%	56.1%	82.6%	76.6%	74.8

On the other hand, the different library preparation protocols resulted in up to 4.5% differences in global methylation levels. The difference was most prominent in the immortalized B-cell line, NA10860, which is intermediary methylated on a global scale (average per library 55.8–60.3%). The highest global methylation levels derived from the NA10860 cell line were observed in the TSDM libraries (59.1–60.3%) and lowest in SPLAT and Accel-NGS Methyl-Seq libraries (55.8%-57.7%) (Fig 4A, S3 Table). Analysis of average methylation in 100 kB windows in the same data further confirmed this pattern (Fig 4B). For the leukemic REH cell-line, the largest inter-library difference observed was 2.6% (average global methylation levels 81.1–82.1% and 80.3–80.6% for SPLAT/Accel-NGS and TSDM libraries, respectively). Hence, we suggest that the variability observed in global methylation levels are more likely caused by alternative factors in the chemistry or PCR amplification used during library preparation than by issues with base calling or Q-scores and may be related to the GC biases observed for some library methods.

### Concordance of methylation calls between HiSeq platforms

**Fig 5:**
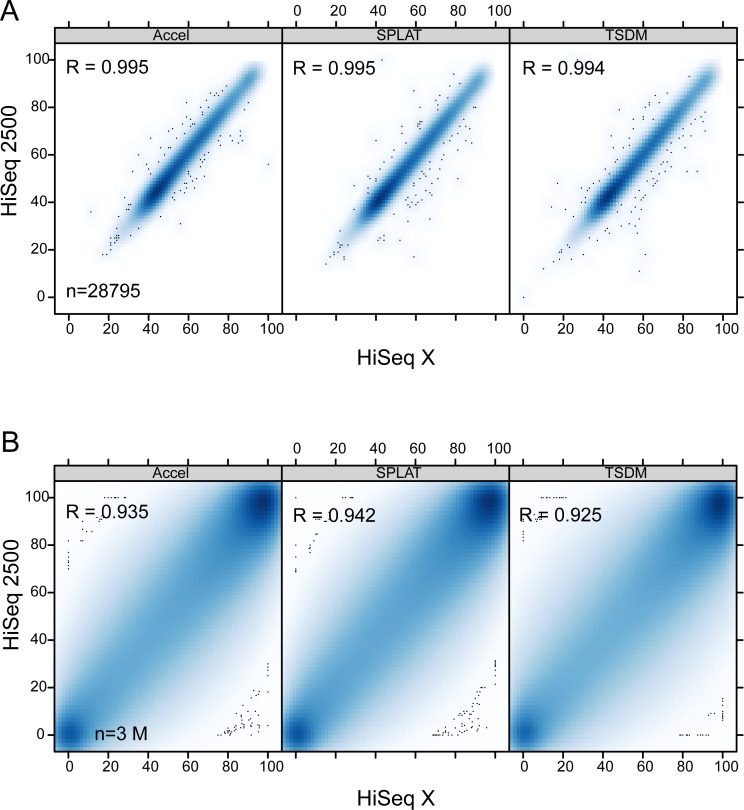
Scatter plots illustrating the high correlation of methylation calls. A) Average methylation in 100 kB windows (n = 28,795) shown for data generated on HiSeq X and HiSeq 2500 systems. B) Methylation at individual CpG sites covered by more than 10 reads (n = 7.5 M) shown for the corresponding data sets.

Next, we analyzed the correlation of methylation calls across the same type of libraries sequenced on HiSeq X and HiSeq 2500, by comparing either individual CpG sites or average methylation in 100 kb non overlapping windows. For methylation in 100 kb windows the Pearson’s correlation coefficient (Pearson’s R) was > 0.99 in comparisons between HiSeq X RTA:2.7.7 and HiSeq 2500 verifying that methylation levels are called with high confidence on the HiSeq X platform (Fig 5A). Similarly, the Pearson’s R was > 0.99 when we compared the average methylation in 100 kB windows between all versions of the HiSeqX and HiSeq2500 (data not shown).

**Fig 6:**
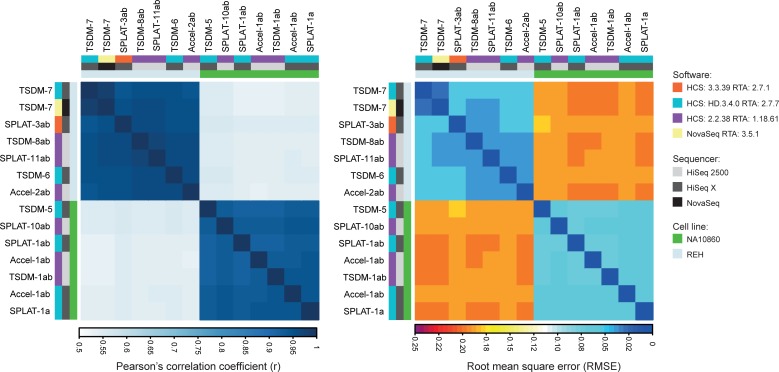
Correlation plots showing pairwise comparisons across a shared set of 3 M CpG sites covered by 10 reads or more in each dataset. A). Heatmap of the Pearson’s correlation coefficients for comparisons across all the library types, sequencing softwares, and cell types used in the study ordered by hierarchical clustering. B) The corresponding root mean square error (RMSE) values for the library/software comparisons in the same order as plotted in panel A.

In order to obtain sufficient coverage to assess correlation at individual CpG sites across the various datasets we combined technical replicates sequenced with the same software versions (SPLAT1a+b, SPLAT3a+b, Accel1a+b, etc, see S1 Fig for an overview). In pairwise comparisons of single CpG sites, only sites covered by >10 reads, in each of the libraries were included. This resulted in the analysis of ~3 M CpG sites in total and thus the correlations were computed across the same set of CpG sites in all comparisons. The correlations varied depending on sample type, however across high Q-score runs (HiSeq X RTA 2.7.7 vs HiSeq2500 RTA 1.18.61), the Pearsons’s R was 0.92–0.94 for intra-library comparisons of the NA10860 sample and 0.96 for the REH sample (Figs 5B and 6A). When comparing SPLAT libraries sequenced with software versions displaying low guanine Q-scores in R2 (SPLAT1ab and SPLAT3ab; RTA2.7.1) to those with high quality scores (SPLAT 1a; RTA2.7.7 and SPLAT10ab, SPLAT11ab; RTA 1.18.61) the Pearson’s R was 0.93 and 0.96 (for NA10860 and REH respectively) and thus in the same range as the high Q-score runs (Fig 6A). The highest correlation was observed for the TSDM-7 library that was sequenced on both HiSeqX (RTA2.7.7) and NovaSeq (Pearson’s R = 0.98). For inter-library comparisons (e.g SPLAT-TSDM, SPLAT-Accel etc) the correlation coefficient was in the same range as the intra-library comparisons (sample NA10860; Pearson’s R = 0.92–0.94), irrespective of software version (Fig 6A). As a second mean to measure the variability of methylation at individual CpG sites across data we computed the pairwise root mean square error (RMSE). The RMSE values were low within the same DNA sample type; 0.031–0.052 for the REH sample and 0.057–0.069 for the NA10860 sample (Fig 6B). The libraries plotted in Fig 6 were ordered based on hierarchical clustering, where we observed co-correlation based on the cell line, but no co-correlation based on sequencing instrument, RTA version, nor library type. Although the number of replicates in this analysis is relatively low, we conclude that low guanine Q-scores in R2 do not have a major impact on the measurement of methylation levels of individual CpG sites across the data sets analyzed herein.

## Conclusion

In this report we compared WGBS data generated with different Illumina platforms and software versions. We found that certain older software versions on the HiSeq X (RTA 2.7.1 and 2.7.5) exhibit severe issues with base quality scoring predominantly in guanines in R2, which represent methylated positions. In such instances where the Q-tables used by the base calling software is not adapted to bisulfite reads it is not possible to determine if a low Q-score value reflects a true uncertainty in the guanine base calling or whether the base quality scores are merely under-estimated. For HiSeq 2500 and HiSeq X RTA2.7.7 the R2 guanine Q-scores are well above 30 and significantly higher than obtained in older versions of HiSeqX. However the R2 guanine Q-scores do remain one of the lowest and most variable bases even with software improvements.

However, as we demonstrate in the present study, when using a popular workflow for WGBS pre-processing, alignment and methylation calling [15] we did not detect any gross methylation bias in those WGBS datasets with low R2 guanine Q-scores as compared to corresponding data with higher Q-scores. Although software-related methylation biases cannot be completely ruled out by our comparison, it is important to note that larger differences in absolute methylation levels were observed across library preparation methods than between different software versions. Nevertheless, we advocate that inspecting base quality scores per nucleotide type for WGBS generated on Illumina systems should be the standard and that sequencer software version should be reported for WGBS data submitted to journals and databases. Importantly, although many WGBS libraries still suffer from short insert sizes and high read duplication levels resulting in comprehensive data loss, with respect to base call quality we find that WGBS data generated with the HD.3.4.0 /RTA 2.7.7 HiSeq X version generated high quality data comparable to those obtained with HiSeq2500, at approximately one fourth of the per base cost.

We also evaluated WGBS data from an installation run on the new NovaSeq system and found that the present Q-scoring is still not optimal for bisulfite sequencing. Despite this, the methylation concordance was very high when comparing to data generated on the HiSeq X system. In our installation run, 50% phiX was spiked-in to evaluate the performance of the machine, but it should be noted that high amounts of phiX or another balanced library is not likely to be required for WGBS on the NovaSeq. Additional data are needed to systematically assess and properly validate how the two-color detection system and the novel Q-score binning approach applied on NovaSeq (using only four Q-score values) reconcile with bisulfite sequencing and downstream analysis.

## Materials and methods

### Library preparation

Human genomic DNA from lymphoblastoid B- cell lines was obtained from the Coriell Institute for Medical Research. Genomic DNA from the pre-B acute lymphoblastoid leukemia cell line REH (was isolated using the AllPrep Universal kit (Qiagen).

The EZ DNA Methylation Gold kit (Zymo Research) was used for sodium bisulfite conversion of DNA prior to library preparation. All WGBS libraries were prepared from 100 ng of genomic DNA. Accel-NGS Methyl-Seq (Swift BioSciences) and TruSeq DNA Methylation (Illumina Inc) were prepared according to the manufacturer’s protocols. SPLAT libraries were prepared as described previously [13] with the exception for libraries SPLAT-8 and SPLAT-9 which were amplified with the Phusion U Hot Start DNA polymerase (Thermo Fisher Scientific), whereas the other SPLAT libraries were amplified using the KAPA HiFi Uracil+ DNA polymerase (KAPA Biosystems).

### Sequencing and data analysis

Paired end sequencing (2 x 150) was performed on a HiSeq X system at the SNP&SEQ Technology Platform. The amount of library loaded on the instrument varied between 100 and 200 pM. A PhiX library was spiked in at 20–40% for HCS v3.3.39 /RTA:2.7.1 or v3.3.75/RTA:2.7.5 and at 2% for HD.3.4.0 /RTA:2.7.7). For comparison we also analyzed previously generated sequencing data from the HiSeq2500 system (HCS 2.2.38 / RTA 1.18.61) using the TruSeq v.4 chemistry PE125 (10% PhiX) [13] and data generated on the an installation run of a NovaSeq 6000 instrument with 50% phiX spike-in (RTA:3.1.5).

Per nucleotide quality scores were extracted using FASTX-Toolkitv 0.0.14 or reported by Sisyphus, an in-house pipeline used at the SNP&SEQ Technology Platform for processing and QC of Illumina sequence data. Sequence reads were quality filtered and adaptors were trimmed using TrimGalore. For Accel-NGS Methyl Seq libraries 18 bp was trimmed off the 5’-end of R2 and the 3’ end of R1 to remove bases derived from the sequence tag introduced in the library preparation procedure. Alignment to the human reference assembly GRCh37 and methylation calling was performed with the Bismark software [15] and the pipeline tool ClusterFlow [16]. For TSDM libraries the initial 6 base pairs of each reads were ignored in the methylation calling procedure, to avoid random priming biases. Global methylation rates (∑C/∑(C+T) in CpG context) and methylation at individual CpG sites was obtained from Bismark methylation extractor output files. Average methylation in 100 kB non overlapping windows were determined using BEDTools. Methylation correlation at individual CpG sites and at 100 kB non overlapping windows and pair-wise root mean square error (RMSE) values were computed using custom R scripts.

## Supporting information

S1 Fig Outline of samples, library pools and software versions used in the comparison. (PDF)

S2 Fig Average guanine Q-scores increases with the library GC content. (PDF)

S3 Fig Base call quality score plots for an Accel-NGS MethylSeq library. (PDF)

S1 Table Per nucleotide quality scores for all sequencing runs performed with RTA 2.7.1 and RTA 2.7.5. (PDF)

S2 Table Per nucleotide quality scores for all sequencing runs performed with RTA 2.7.7. (PDF)

S3 Table Global methylation levels per library and software versions. (PDF)

## References

[1] Jones PA. Functions of DNA methylation: islands, start sites, gene bodies and beyond. Nature reviews Genetics. 2012;13(7):484–92. Epub 2012/05/30. doi: 10.1038/nrg3230 . 22641018 10.1038/nrg3230

[2] Portela A, Esteller M. Epigenetic modifications and human disease. Nature biotechnology. 2010;28(10):1057–68. Epub 2010/10/15. doi: 10.1038/nbt.1685 . 20944598 10.1038/nbt.1685

[3] Frommer M, McDonald LE, Millar DS, Collis CM, Watt F, Grigg GW, et al A genomic sequencing protocol that yields a positive display of 5-methylcytosine residues in individual DNA strands. Proceedings of the National Academy of Sciences of the United States of America. 1992;89(5):1827–31. Epub 1992/03/01. ; PubMed Central PMCID: PMCPmc48546. 1542678 10.1073/pnas.89.5.1827 PMC48546

[4] Moran S, Arribas C, Esteller M. Validation of a DNA methylation microarray for 850,000 CpG sites of the human genome enriched in enhancer sequences. Epigenomics. 2016;8(3):389–99. Epub 2015/12/18. doi: 10.2217/epi.15.114 ; PubMed Central PMCID: PMCPmc4864062. 26673039 10.2217/epi.15.114 PMC4864062

[5] Kunze S. Quantitative Region-Specific DNA Methylation Analysis by the EpiTYPER Technology. Methods in molecular biology (Clifton, NJ). 2018;1708:515–35. Epub 2017/12/11. doi: 10.1007/978-1-4939-7481-8_26 . 29224161 10.1007/978-1-4939-7481-8_26

[6] Flusberg BA, Webster DR, Lee JH, Travers KJ, Olivares EC, Clark TA, et al Direct detection of DNA methylation during single-molecule, real-time sequencing. Nature methods. 2010;7(6):461–5. Epub 2010/05/11. doi: 10.1038/nmeth.1459 ; PubMed Central PMCID: PMCPmc2879396. 20453866 10.1038/nmeth.1459 PMC2879396

[7] Simpson JT, Workman RE, Zuzarte PC, David M, Dursi LJ, Timp W. Detecting DNA cytosine methylation using nanopore sequencing. Nature methods. 2017;14(4):407–10. Epub 2017/02/22. doi: 10.1038/nmeth.4184 . 28218898 10.1038/nmeth.4184

[8] Krueger F, Andrews SR, Osborne CS. Large Scale Loss of Data in Low-Diversity Illumina Sequencing Libraries Can Be Recovered by Deferred Cluster Calling. PLOS ONE. 2011;6(1):e16607 doi: 10.1371/journal.pone.0016607 21305042 10.1371/journal.pone.0016607 PMC3030592

[9] Mitra A, Skrzypczak M, Ginalski K, Rowicka M. Strategies for achieving high sequencing accuracy for low diversity samples and avoiding sample bleeding using illumina platform. PLoS One. 2015;10(4):e0120520 Epub 2015/04/11. doi: 10.1371/journal.pone.0120520 ; PubMed Central PMCID: PMCPmc4393298. 25860802 10.1371/journal.pone.0120520 PMC4393298

[10] Illumina. Low Diversity Sequencing on the Illumina HiSeq Platform. Illumina Technical Note. 2014.

[11] Toh H, Shirane K, Miura F, Kubo N, Ichiyanagi K, Hayashi K, et al Software updates in the Illumina HiSeq platform affect whole-genome bisulfite sequencing. BMC genomics. 2017;18(1):31 Epub 2017/01/07. doi: 10.1186/s12864-016-3392-9 ; PubMed Central PMCID: PMCPmc5217569. 28056787 10.1186/s12864-016-3392-9 PMC5217569

[12] Miura F, Enomoto Y, Dairiki R, Ito T. Amplification-free whole-genome bisulfite sequencing by post-bisulfite adaptor tagging. Nucleic acids research. 2012;40(17):e136 Epub 2012/06/01. doi: 10.1093/nar/gks454 ; PubMed Central PMCID: PMCPmc3458524. 22649061 10.1093/nar/gks454 PMC3458524

[13] Raine A, Manlig E, Wahlberg P, Syvanen AC, Nordlund J. SPlinted Ligation Adapter Tagging (SPLAT), a novel library preparation method for whole genome bisulphite sequencing. Nucleic acids research. 2017;45(6):e36 Epub 2016/12/03. doi: 10.1093/nar/gkw1110 ; PubMed Central PMCID: PMCPmc5389478. 27899585 10.1093/nar/gkw1110 PMC5389478

[14] Rosenfeld C, Goutner A, Venuat AM, Choquet C, Pico JL, Dore JF, et al An effect human leukaemic cell line: Reh. European journal of cancer. 1977;13(4–5):377–9. Epub 1977/04/01. . 194778 10.1016/0014-2964(77)90085-8

[15] Krueger F, Andrews SR. Bismark: a flexible aligner and methylation caller for Bisulfite-Seq applications. Bioinformatics (Oxford, England). 2011;27(11):1571–2. Epub 2011/04/16. doi: 10.1093/bioinformatics/btr167 ; PubMed Central PMCID: PMCPmc3102221. 21493656 10.1093/bioinformatics/btr167 PMC3102221

[16] Ewels P, Krueger F, Kaller M, Andrews S. Cluster Flow: A user-friendly bioinformatics workflow tool. F1000Research. 2016;5:2824 Epub 2017/05/20. doi: 10.12688/f1000research.10335.2 ; PubMed Central PMCID: PMCPmc5310375.2. 28299179 10.12688/f1000research.10335.1 PMC5310375

