# Supplementary material for: Data quality of whole genome bisulfite sequencing on Illumina platforms
Source: PLoS One. 2018 Apr 18;13(4):e0195972. doi: 10.1371/journal.pone.0195972 (PMC5905984; doi:10.1371/journal.pone.0195972)
Supplement: S1 Fig — (PDF) [file pone.0195972.s001.pdf]

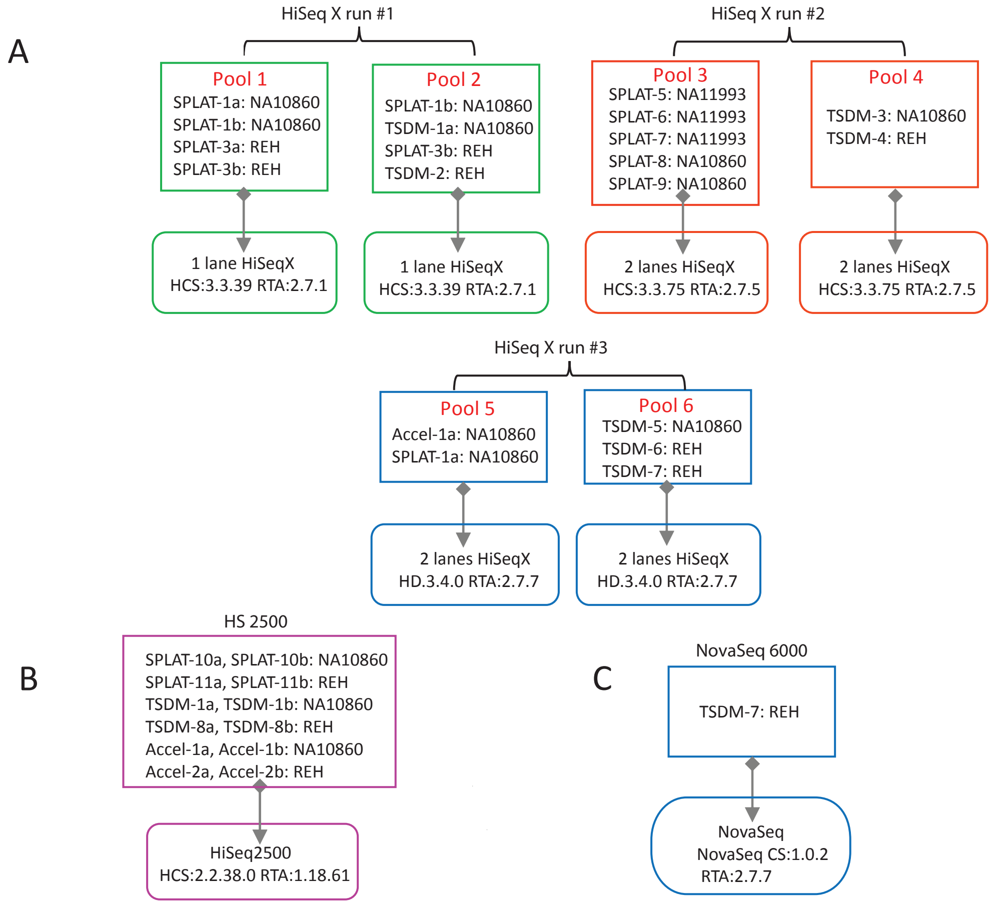

Supplementary Figure 1. Outline of samples, library pools and software versions used in the comparison. A) Libraries sequenced on HiSeq X. For each HiSeq X sequencing run the library pooling was performed with the objective to obtain raw reads corresponding to 30x per cell line. B) Libraries sequenced on HiSeq 2500. C) Libraries sequenced on NovaSeq.
