# Supplementary material for: Data quality of whole genome bisulfite sequencing on Illumina platforms
Source: PLoS One. 2018 Apr 18;13(4):e0195972. doi: 10.1371/journal.pone.0195972 (PMC5905984; doi:10.1371/journal.pone.0195972)
Supplement: S2 Fig — (PDF) [file pone.0195972.s002.pdf]

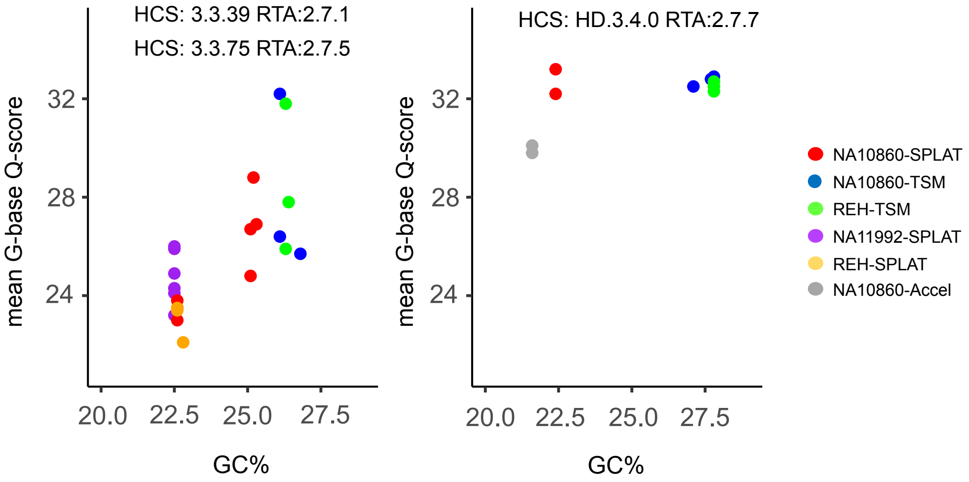

Supplementary Figure 2. Average guanine Q-scores increases with the library GC content. For HiSeq X software versions RTA 2.7.1 and 2.7.7, a trend towards higher Q-scores with increasing GC content was observed. This trend was not apparent in data generated with RTA 2.7.7.
