# Supplementary material for: Data quality of whole genome bisulfite sequencing on Illumina platforms
Source: PLoS One. 2018 Apr 18;13(4):e0195972. doi: 10.1371/journal.pone.0195972 (PMC5905984; doi:10.1371/journal.pone.0195972)
Supplement: S3 Fig — (PDF) [file pone.0195972.s003.pdf]

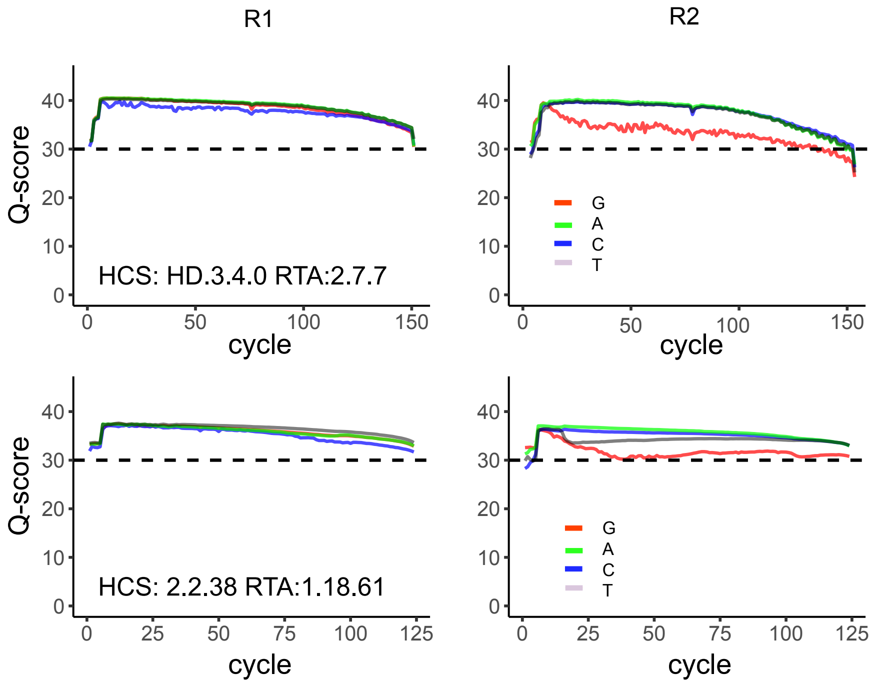

Supplementary Figure 3. Base call quality score plots for an Accel-NGS Methyl-Seq library. Per nucleotide Q-score plots for an Accel-NGS Methyl-Seq library (cell line NA10860) sequenced both on HiSeq X (RTA 2.7.7) and HiSeq 2500 (RTA 1.18.61)
