# Supplementary material for: Data quality of whole genome bisulfite sequencing on Illumina platforms
Source: PLoS One. 2018 Apr 18;13(4):e0195972. doi: 10.1371/journal.pone.0195972 (PMC5905984; doi:10.1371/journal.pone.0195972)
Supplement: S1 Table — (PDF) [file pone.0195972.s004.pdf]

Software version	Library	Sample	Read 1 Q30/base Lane 1	Read 2 Q30/base Lane 1	Read 1 Q30/base Lane 2	Read 2 Q30/base Lane 2
HCS: 3.3.39<br>RTA:2.7.1	SPLAT-1	NA10860	A:37.3±8.3<br>C:33.8±10.8<br>G:37.2±8.5<br>T:36.8±8.7	A:29.6±11.4<br>C:32.7±10.6<br>G:23.0±11.7<br>T:32.0±11.0	n.d	n.d
	SPLAT-2	NA10860	A:37.2±8.4<br>C:34.0±10.6<br>G:37.1±8.5<br>T:36.7±8.8	A:29.9±11.4<br>C:32.9±10.6<br>G:23.5±11.7<br>T:32.1±11.0	A:37.5±8.0<br>C:35.8±9.6<br>G:37.7±7.9<br>T:37.1±8.3	A:30.1±11.4<br>C:33.0±10.6<br>G:23.8±12.0<br>T:31.3±11.2
	SPLAT-3	REH	A:36.6±9.0<br>C:32.0±11.6<br>G:36.5±9.0<br>T:36.1±9.3	A:28.2±11.7<br>C:31.4±11.0<br>G:22.1±11.5<br>T:30.7±11.4	n.d	n.d
	SPLAT-4	REH	A:37.4±8.3<br>C:34.1±10.5<br>G:37.3±8.4<br>T:36.9±8.6	A:29.9±11.5<br>C:32.7±10.7<br>G:23.5±11.7<br>T:32.1±11.0	A:37.3±8.2<br>C:35.2±9.9<br>G:37.4±8.2<br>T:36.9±8.5	A:29.7±11.5<br>C:32.5±10.8<br>G:23.4±12.0<br>T:30.9±11.3
	TSDM-1	NA10860	A:36.1±9.2<br>C:35.0±10.2<br>G:35.7±8.9<br>T:33.1±10.3	A:28.8±11.8<br>C:32.6±10.9<br>G:25.7±12.0<br>T:30.8±11.5	n.d	n.d
	TSDM-2	REH	A:37.6±8.3<br>C:36.0±9.8<br>G:38.3±7.5<br>T:37.4±8.2	A:30.6±11.5<br>C:34.3±10.1<br>G:27.8±11.8<br>T:32.6±10.9	n.d	n.d
HCS: 3.3.75<br>RTA:2.7.5	SPLAT-5	NA11992	A:37.0±7.5<br>C:34.8±9.5<br>G:36.6±8.1<br>T:36.6±7.9	A:32.9±10.0<br>C:33.8±9.9<br>G:24.9±11.6<br>T:33.0±10.2	A:36.9±7.6<br>C:34.3±9.8<br>G:36.5±8.1<br>T:36.7±7.7	A:30.9±10.6<br>C:32.7±10.2<br>G:23.2±10.9<br>T:32.3±10.4
	SPLAT-6	NA11992	A:37.6±7.0<br>C:36.2±8.3<br>G:37.3±7.4<br>T:37.3±7.3	A:33.8±9.7<br>C:34.5±9.6<br>G:26.0±11.5<br>T:34.0±9.7	A:37.5±7.1<br>C:35.8±8.5<br>G:37.1±7.4<br>T:37.3±7.	A:31.9±10.3<br>C:33.6±9.8<br>G:24.1±10.9<br>T:33.3±10.0
	SPLAT-7	NA11992	A:37.5±7.1<br>C:36.1±8.4<br>G:37.3±7.4<br>T:37.2±7.4	A:33.5±9.9<br>C:34.2±9.8<br>G:25.9±11.5<br>T:33.6±10.0	A:37.5±7.1<br>C:35.8±8.5<br>G:37.1±7.4<br>T:37.3±7.2	A:31.7±10.4<br>C:33.3±9.9<br>G:24.3±10.9<br>T:32.9±10.2
	SPLAT-8	NA10860	A:37.7±6.9<br>C:35.7±9.0<br>G:37.5±7.2<br>T:37.1±7.5	A:34.2±9.4<br>C:35.4±8.9<br>G:28.8±11.0<br>T:34.6±9.4	A:37.5±7.0<br>C:35.4±9.2<br>G:37.3±7.4<br>T:37.0±7.4	A:32.1±10.2<br>C:34.3±9.3<br>G:26.9±10.7<br>T:33.7±9.7
	SPLAT-9	NA10860	A:37.5±7.0<br>C:36.4±8.1<br>G:37.5±7.2<br>T:37.1±7.5	A:33.5±9.8<br>C:34.4±9.6<br>G:26.7±11.4<br>T:33.7±9.9	A:37.5±7.0<br>C:36.1±8.1<br>G:37.3±7.2<br>T:37.1±7.4	A:31.5±10.4<br>C:33.5±9.8<br>G:24.8±10.8<br>T:33.0±10.1
	TSDM-3	NA10860	36.1±9.2<br>35.0±10.2<br>35.7±8.9<br>33.1±10.3	28.8±11.8<br>32.6±10.9<br>25.7±12.0<br>30.8±11.5	n.d	n.d
	TSDM-4	REH	37.6±8.3<br>36.0±9.8<br>38.3±7.5<br>37.4±8.2	30.6±11.5<br>34.3±10.1<br>27.8±11.8<br>32.6±10.9	n.d	n.d

Supplementary Table 1. Per nucleotide quality scores for all sequencing runs performed with HCS 3.3.39/ RTA 2.7.1 and HCS 3.3.75/ RTA 2.7.5.
