# Supplementary material for: Data quality of whole genome bisulfite sequencing on Illumina platforms
Source: PLoS One. 2018 Apr 18;13(4):e0195972. doi: 10.1371/journal.pone.0195972 (PMC5905984; doi:10.1371/journal.pone.0195972)
Supplement: S2 Table — (PDF) [file pone.0195972.s005.pdf]

Software version	Library	Sample	Read 1 Q30/base Lane 1	Read 2 Q30/base Lane 1	Read 1 Q30/base Lane 2	Read 2 Q30/base Lane 2	Read 1 Q30/base Lane 3	Read 2 Q30/base Lane 3
HCS: HD.3.4.0<br>RTA:2.7.7	Accel-1	NA10860	A:38.4±6.3<br>C:37.0±8.4<br>G:37.8±7.3<br>T:38.3±6.2	A:33.8±9.9<br>C:34.6±9.3<br>G:30.1±11.5<br>T:33.5±9.9	A:38.1±6.8<br>C:36.4±9.1<br>G:37.4±7.8<br>T:37.9±6.7	A:32.8±10.3<br>C:33.9±9.6<br>G:29.8±11.5<br>T:32.6±10.2	n.d	n.d
	SPLAT-1	NA10860	A:38.6±5.8<br>C:36.1±9.0<br>G:38.3±6.4<br>T:38.4±6.2	A.38.2±6.4<br>C:38.1±6.7<br>G.33.2±10.8<br>T:37.9±6.9	A:38.5±6.0<br>C:36.2±9.0<br>G:38.0±6.7<br>T:38.2±6.4	A:37.9±6.7<br>C:37.8±7.0<br>G:32.2±11.1<br>T:37.5±7.3	n.d	n.d
	TSDM-5	NA10860	A:38.1±6.5<br>C:35.6±9.5<br>G:37.8±7.0<br>t:38.0±6.7	A:37.8±7.0<br>C.37.5±7.4<br>G:32.5±11.2<br>T:37.0±8.0	A:38.1±6.5<br>C:35.5±9.6<br>G:37.7±7.1<br>T:38.0±6.7	A:38.0±6.7<br>C:37.6±7.2<br>G:32.8±11.3<br>T:37.4±7.6	A:38.4±6.2<br>C:35.4±9.8<br>G:38.1±6.7<br>T:38.2±6.4	A:38.1±6.7<br>C.37.7±7.1<br>G:32.9±11.5<br>T:37.4±7.6
	TSDM-6	REH	A:38.0±6.6<br>C:35.6±9.4<br>G:37.5±7.3<br>T.37.8±6.9	A:37.6±7.2<br>C:37.3±7.5<br>G:32.3±11.2<br>T:36.8±8.2	A:38.0±6.5<br>C:35.6±9.4<br>G:37.5±7.3<br>T:37.9±6.9	A:37.8±7.0<br>C:37.4±7.4<br>G:32.5±11.4<br>T:37.1±7.9	A:37.8±7.0<br>C:37.4±7.4<br>G:32.5±11.4<br>T:37.1±7.9	A:37.8±6.9<br>C:37.5±7.4<br>G:32.7±11.4<br>T:37.1±7.9
	TSDM-7	REH	37.3±7.3<br>35.2±9.6<br>36.7±8.1<br>37.3±7.5	37.6±7.2<br>37.3±7.6<br>31.6±11.6<br>36.7±8.4	37.2±7.6<br>34.8±9.8<br>36.5±8.4<br>37.1±7.7	37.4±7.5<br>37.1±7.7<br>30.8±12.0<br>36.5±8.6	37.8±6.9<br>35.4±9.6<br>37.4±7.6<br>37.7±7.1	A:37.0±7.9<br>C:36.7±8.2<br>G:30.5±12.3<br>T:36.0±9.0

Supplementary Table 2. Per nucleotide quality scores for all sequencing runs performed with HCS HD 3.4.0/ RTA 2.7.7
