# Supplementary material for: Data quality of whole genome bisulfite sequencing on Illumina platforms
Source: PLoS One. 2018 Apr 18;13(4):e0195972. doi: 10.1371/journal.pone.0195972 (PMC5905984; doi:10.1371/journal.pone.0195972)
Supplement: S3 Table — (PDF) [file pone.0195972.s006.pdf]

Library / Sample	RTA version	Global methylation (%)
SPLAT-1a NA10860	2.7.7	55.8
SPLAT-1a NA10860	2.7.1	56.0
SPLAT-1b NA10860	2.7.1	56.1
SPLAT-10a NA10860	1.18.61	57.4
SPLAT-10b-NA10860	1.18.61	56.8
SPLAT-8-NA10860	2.7.5	57.8
SPLAT-9-NA10860	2.7.5	57.5
Accel-1a NA10860	2.7.7	57.7
Accel-1a NA10860	1.18.61	57.6
Accel-1b NA10860	1.18.61	57.5
TSDM-5 NA10860	2.7.7	60.3
TSDM-1a NA10860	2.7.1	59.5
TSDM-1a NA10860	1.18.61	59.4
TSDM-1b NA10860	1.18.61	59.2
TSDM-3 NA10860	2.7.5	59.1
SPLAT-3a REH	2.7.1	81.3
SPLAT-3b REH	2.7.1	81.1
SPLAT-11a REH	1.18.61	82.3
SPLAT-11b REH	1.18.61	81.8
Accel-2a REH	1.18.61	82.5
Accel-2b REH	1.18.61	82.7
TSDM-6 REH	2.7.7	80.6
TSDM-7 REH	2.7.7	81.2
TSDM-7 REH	3.1.5	81.2
TSDM-2 REH	2.7.1	80.6
TSDM-4 REH	2.7.5	80.2
TSDM-8a REH	1.18.61	80.3
TSDM-8a REH	1.18.61	80.4

Supplementary Table 3. Global methylation levels per library and software version
